# CYP450 core involvement in multiple resistance strains of *Aedes aegypti* from French Guiana highlighted by proteomics, molecular and biochemical studies

**DOI:** 10.1371/journal.pone.0243992

**Published:** 2021-01-11

**Authors:** Yanouk Epelboin, Lanjiao Wang, Quentin Giai Gianetto, Valérie Choumet, Pascal Gaborit, Jean Issaly, Amandine Guidez, Thibaut Douché, Thibault Chaze, Mariette Matondo, Isabelle Dusfour

**Affiliations:** 1 Unité d’Entomologie Médicale, Institut Pasteur de la Guyane, Cayenne, French Guiana, France; 2 Proteomics Platform, Mass Spectrometry for Biology Unit, USR CNRS 2000, Institut Pasteur, Paris, France; 3 Bioinformatics and Biostatistics HUB, Computational Biology Department, USR CNRS 3756, Institut Pasteur, Paris, France; 4 Environment and Infectious risks Unit, Institut Pasteur, Paris, France; 5 Global Health department, Institut Pasteur, Paris, France; Institute of Zoology Chinese Academy of Sciences, CHINA

## Abstract

Insecticide resistance is a worldwide threat for vector control around the world, and *Aedes aegypti*, the main vector of several arboviruses, is a particular concern. To better understand the mechanisms of resistance, four isofemale strains originally from French Guiana were isolated and analysed using combined approaches. The activity of detoxification enzymes involved in insecticide resistance was assayed, and mutations located at positions 1016 and 1534 of the sodium voltage-gated channel gene, which have been associated with pyrethroid resistance in *Aedes aegypti* populations in Latin America, were monitored. Resistance to other insecticide families (organophosphates and carbamates) was evaluated. A large-scale proteomic analysis was performed to identify proteins involved in insecticide resistance. Our results revealed a metabolic resistance and resistance associated with a mutation of the sodium voltage-gated channel gene at position 1016. Metabolic resistance was mediated through an increase of esterase activity in most strains but also through the shifts in the abundance of several cytochrome P450 (CYP450s). Overall, resistance to deltamethrin was linked in the isofemale strains to resistance to other class of insecticides, suggesting that cross- and multiple resistance occur through selection of mechanisms of metabolic resistance. These results give some insights into resistance to deltamethrin and into multiple resistance phenomena in populations of *Ae*. *aegypti*.

## 1. Introduction

*Aedes aegypti* is an anthropophilic, endophagic and endophilic mosquito species [1], which can transmit several arboviruses. Extensive sprawling urbanization and the increasing human population have facilitated geographical expansion of *Ae*. *aegypti* populations around the world due in part to proliferation of breeding sites [2]. In parallel, a dramatic number of arbovirus outbreaks, including dengue, Zika and chikungunya viruses has risen worldwide. In the absence of effective vaccines, preventing *Ae*. *aegypti* bites by reducing densities and vector-human contacts remains the major mean for reducing disease burden. Several strategies are used such as governmental vector control programs, enhanced community engagement or individual protection campaigns. Historically, reducing density is mainly performed by breeding sites removal and treatment along with spatial insecticide spraying against adult mosquitoes [3]. Organophosphates and pyrethroids are insecticides families that have long been used to target adults, especially during outbreak. However, due to their toxicity, organophosphates were banned in the European Union in 2011, leaving deltamethrin one of the only available compounds to be used against adult mosquitoes.

French Guiana is an overseas French territory and is under European regulation concerning vector control. Malathion, temephos (organophosphates) and deltamethrin (pyrethroid) have been used for many years for the control of *Ae*. *aegypti* leading to resistance to those insecticides, particularly deltamethrin [4].

High resistance to deltamethrin in *Ae*. *aegypti* populations has been described worldwide, compromising the effectiveness of spraying operations [5, 6]. Underlying resistance mechanisms are multiple and include behavioural and/or physiological changes in mosquitoes leading to insecticide avoidance, altered penetration and sequestration, which are poorly studied [7], target site modification and metabolic resistance which are both the most studied mechanisms. Deltamethrin, like other type II pyrethroids, targets the nervous system, specifically voltage-dependent sodium channels, and disturbs channel-opening kinetics, leading to paralysis and the death of the insect [8]. In mosquitoes, pyrethroid resistance is mainly associated with mutations in the gene that codes for the voltage-gated sodium channel protein, leading to knock-down resistance (*kdr*) [9]. In American populations of *Ae*. *aegypti*, resistance to pyrethroids is closely linked to substitutions in the voltage-gated sodium channel protein, particularly valine to isoleucine at the 1016 site (V1016I) and phenylalanine to cysteine at the 1534 site (F1534C) [4, 10, 11]. Other point mutations of this channel have been described in *Aedes* mosquitoes from Asia or in *Anopheles* mosquitoes and may lead to pyrethroid resistance in these populations [12, 13]. The enzymatic mechanisms responsible for insecticide resistance include detoxifying enzymes such as cytochrome P450 mixed-function oxidases which are the largest class of mixed-function oxidases (MFO) and non-specific esterases [9]. Esterases are generally associated with resistance to organophosphates, particularly to temephos [14, 15], and MFO are involved in pyrethroid resistance [14, 16, 17].

Multiple resistance may occur when the use of one insecticide leads to the development of resistance against other insecticides from the same or other classes [16, 18]. Consequently, the mechanisms of resistance to new molecules become difficult to anticipate, and the selection of alternative molecules with different modes of action becomes strenuous to obtain for managing resistance and guarantee population control efficacy.

Knowledge of the underlying mechanisms as well as the assessment and the surveillance of reliable markers are mandatory to anticipate insecticide resistance [19]. Our study follows this line with the objective to highlight the mechanisms used by *Ae*. *aegypti* to resist multiple insecticides. For this, we performed bioassays, enzymatic assays and mass spectrometry-based (MS) proteomics from multi-resistant laboratory strains. Our results provide new and complementary information to the transcriptomic approaches used until now to characterize resistance pattern in French Guiana [11, 20–22]. Those results will provide baseline for the vector competence and resistance studies.

## 2. Methods

### 2.1. Ethics statement

Anesthetized mice were used as the blood source for feeding mosquitoes and maintaining mosquito stain, rabbits’ fresh blood was applied for preparing the artificial meal with virus to infect target mosquitoes. Experiments were authorized by the agreement number B973-02-01 delivered by "La préfecture de Guyane" and renewed on June 6th, 2015. The protocol for the utility of mice and rabbits was approved by the ethical committee CETEA Institut Pasteur (n° 89), report number 2015–0010 issued on May 18th, 2015.

### 2.2. Mosquito strains

Six *Ae*. *aegypti* strains were used. The parent population was obtained from larvae collected in an area with low insecticide pressure, Île Royale (5.287° N; 52.590° W), an island in the Atlantic Ocean off the coast of French Guiana. The initial field-collected population in 2015 was labelled IR0115 and maintained in parallel in laboratory conditions without selection treatment until F_11_. Four isofemale strains (inbred lines of mosquitoes derived from the progeny of one isolated mated female) were isolated. Two that showed relatively low resistance to deltamethrin were reared until F_9_ (labelled IR13 and IR36) with no exposure to deltamethrin. The other two strains, which showed stronger resistance, were reared until F_10_ (labelled IR03 and IR05) without treatment; then, from F_10_, their resistance to deltamethrin was accumulated by eliminating 70% of larvae by exposure to deltamethrin every two generations until F_19_. The susceptible reference strain New Orleans (NO) was maintained regularly in laboratory conditions for World Health Organization (WHO) bioassays or as the negative control for biochemical assays. NO was also chosen to establish a baseline of diagnostic concentrations for 9 insecticides. Only IR03 F_20_, IR05 F_20_ and IR13 F_11_, as well as NO strain, were used for proteomic analyses.

### 2.3. Mosquito rearing and processing

Field-collected larvae and pupae were placed in the insectary at the Pasteur Institute of French Guiana. Larvae were fed on brewer’s yeast tablets (Gayelord Hauser) until the pupal stages. Pupae were collected daily and transferred to screened holding cages (30 x 30 x 30 cm). Adult mosquitoes were fed on cotton soaked in 10% sugar. To obtain a new generation of mosquitoes, 3–4-day-old females fed on anaesthetized mice; the oviposition site was a laying cup with paper wick placed in the cages. The eggs were conserved appropriately for a maximum of 6 months before starting the next generation. The rearing conditions included a daily temperature of 28 ± 2°C, a relative humidity of 80 ± 5% and a 12:12 h light:dark phase. For proteomic analyses, 5-day-old females of the four strains (IR03, IR05, IR13 and NO) were starved 36 h before the blood feeding. Mosquitoes fed 30 minutes on an artificial membrane feeding system HEMOTEK (6W1, Hemotek Ltd, UK) with washed rabbit erythrocytes.

### 2.4. Insecticides

Technical grades of deltamethrin (#45423, Sigma-Aldrich), alpha-cypermethrin (#45806, Sigma-Aldrich), permethrin (#45614, Sigma-Aldrich), malathion (#45614, Sigma-Aldrich), fenitrothion (#45487, Sigma-Aldrich), chlorpyrifos-methyl (#45396, Sigma-Aldrich), propoxur (#45644, Sigma-Aldrich), bendiocarb (#45336, Sigma-Aldrich) and fipronil (#46451, Sigma-Aldrich) were used in the experiments. A series of dilutions of each insecticide that caused the death of 0–100% of 3–5-day-old adult females were prepared and impregnated on filter papers according to the WHO procedure [23]. Briefly, 2 mL of the desired concentration of the selected insecticide solubilized in acetone and mineral oil were introduced onto a sheet of filter paper (Whatman no. 1, 15 x 12 cm). The papers were then dried overnight and wrapped in aluminium foil. The control paper was impregnated with only acetone and mineral oil.

### 2.5. Insecticide assays

#### 2.5.1. Susceptibility baselines

The diagnostic concentrations of the nine selected insecticides were established in dose–response assays on the *Ae*. *aegypti* NO susceptible reference strain. For all chemicals, five doses were used to establish the baseline 50% and 99% lethal concentrations (LC_50_ and LC_99_). Bioassays were performed into standard tubes [23]. Twenty-five 3–5-day-old female mosquitoes, sugar fed, were sorted on ice (4°C), introduced into a holding tube and left for 1 h at ambient temperature (28 ± 2°C) to allow the mosquitoes to revive from the cold shock. Mosquitoes that had recovered well were then transferred into the testing tubes and exposed for 1 h to the insecticide-impregnated papers. They were then transferred back into the holding tubes and provided with cotton soaked in 10% sugar solution. The mortality rate was observed after 24 h of incubation without insecticides at room temperature (28 ± 2°C). Each condition was tested in four replicates and with two negative controls. The diagnostic concentrations were determined from the values corresponding to twice the LC99.

#### 2.5.2. Susceptibility test on selected laboratory strains

Bioassays were performed on all strains including NO, as described previously, with the established diagnostic doses of each insecticide. The susceptibility of *Ae*. *aegypti* populations (24 h mortality) was determined according to WHO recommendations [24]: a mortality rate of ≥ 98% characterizes a population as *susceptible*; < 90% as *confirmed resistant*; and 90–97% as *possibly resistant*. In the last case, the test was duplicated; if the mortality rate was still < 98%, the presence of resistance was confirmed [24].

### Knockdown resistance (*kdr*) genotyping

#### 2.6.1. DNA extraction

Genomic DNA was extracted individually from at least three legs of adult mosquito according to the protocol of Collins et al. [25].

#### 2.6.2. Discrimination allele assays

Discrimination allele assays for V1016I and F1534C were conducted with a StepOnePlus Real-time PCR system (Life Technologies) as previously described [11, 26]. The allelic discrimination assay for V1016I was composed of two standard oligonucleotides, V1016I SNP-F-GCT-AAC-CGA-CAA-ATT-GTT-TCC-C and V1016I SNP-R-CAG-CGA-GGATGA-ACC-GAA-AT. The two probes consist of a 5´ reporter dye, a 3´ non-fluorescence quencher and a minor groove binder at the 3´ end. The probe V1016-PV CAC-AGG-TAC-TTA-ACCTTT-T was labelled with 6-Vic dye fluorescence at the 5´ end for detection of the wild-type allele, and the probe I1016-PF CAC-AGA-TAC-TTA-ACC-TTT-TC was labelled with FAM dye fluorescence at the 5´ end for detection of the mutant allele.

The allelic discrimination assay for F1534C was composed of two standard oligonucleotides, F1534C SNP-F-GAT-GAT-GAC-ACC-GAT-GAA-CAG-ATC and F1534C SNP-R-CGA-GAC-CAA-CAT-CTA-GTA-CCT. The two probes consist of a 5´ reporter dye, a 3´ non-fluorescence quencher and a minor groove binder at the 3´end. The probe F1534-PV AAC-GAC-CCG-AAG-ATG-A was labelled with 6-Vic dye fluorescence at the 5´ end for detection of the wild-type allele, whereas the probe C1534-PF ACGACC-CGC-AGA-TGA was labelled with FAM dye fluorescence at the 5´ end for detection of the mutant allele.

The PCR mix comprised 12.5 μL of Taqman genotyping Master Mix (Applied Biosystems, USA), 1.8 μL of 10 μM reverse primer, 1.8 μL of 10 μM forward primers, 0.5 μL of 10 μM probe for allele wild-type and 0.5 μL of 10 μM probe for allele mutant, 0.9 μL of H_2_O and 3 μL of DNA sample. The thermocycling conditions were: a 95°C, 10-min holding stage, 45 cycles at 95°C for 15 s, a 60°C, 1 min cycling stage and a 60°C, 1-min post-read stage. Data were analysed directly with StepOne Software version 2.3.

### 2.7. Biochemical analyses

#### 2.7.1. Protein extraction

Total protein was extracted from individual mosquitoes homogenized at 4°C with a pestle in 500 μL of 0.01M phosphate buffered saline at pH 7.2 containing protease inhibitors (one tablet of complete EDTA-free protease inhibitor cocktail [Roche] in 25 mL). Solubilized proteins were extracted by centrifugation at 10 000 x *g* for 15 min at 4°C to eliminate cellular debris. The resulting lysates were aliquoted and stored at –80°C for further analysis. The protein content of each lysate was measured with the DC protein assay (Bio-Rad) in 96-well microplates (Nunc) with a Multiskan FC microplate reader (Thermo Scientific) and analysed with Skanlt software v5.0.

#### 2.7.2. Enzymatic assays

The enzyme activity was assayed in mosquitoes not exposed to insecticides.

The activities of MFO and non-specific alpha- and beta-esterases were measured according to the procedures of Valle et al. [27]. Briefly, 20 μL of total protein lysate was used for each enzyme on a separate microplate.

MFO activity was measured by the haem peroxidation method, with 3,3´,5,5´-tetramethylbenzidine (Sigma Aldrich) as the substrate. The method provides indirect estimates of the levels of haem-containing enzymes in individual female mosquitoes, which are then converted into equivalent units of cytochrome P450. The reaction was conducted with 20 μL of total protein lysates, incubated for 20 min in buffer containing 0.05% tetramethylbenzidine diluted in 5 mL acetone in 250 mM sodium acetate buffer (pH 5). Absorbance was read at 620 nm after incubation for 5 min with 25 μL of 3% hydrogen peroxide (Sigma-Aldrich). Concentrations were calculated from a standard curve of cytochrome C obtained from bovine heart (Sigma Aldrich). MFO activity was reported as the amount of cytochrome P450 oxidase and expressed in nmol of P450 equivalent units per mg of protein.

Non-specific esterase activity was measured with two substrates, alpha- and beta-naphthyl acetate. For both assays, 200 μL of alpha- or beta-naphthyl acetate 0.7 mM was added to 20 μL of total protein lysates and incubated at room temperature for 20 min. The reaction was stopped by adding 40 μL of Fast blue B salt buffer (0.3% Fast Blue B salt [Sigma Aldrich, St Louis, MO] / 3.5% sodium dodecyl sulfate in distilled water). The reaction was incubated for 5 min at room temperature, and absorbance was read at 620 nm for alpha-esterase and at 570 nm for beta-esterase. Values were then calculated from a standard curve of alpha- or beta-naphthol. Esterase activity was expressed in units per mg of protein (U/mg) corresponding to the production of 1 μmol of alpha- or beta-naphthol per min per mg of protein.

Total superoxide dismutase (SOD; EC 1.15.1.1) activity was determined with an SOD assay kit (Sigma-Aldrich) according to the manufacturer’s instructions, in which 200 μL of water-soluble tetrazolium salt were added to 20 μL of total protein lysates, and the reaction was initiated by adding 20 μL of a mixture of xanthine oxidase and xanthine. Absorbance of samples was read after incubation at 24°C for 20 min at 450 nm. A standard inhibition curve was created with SOD from bovine erythrocytes. SOD activity was expressed in units per mg of protein (U/mg), 1 U SOD being defined as the amount of enzyme that inhibited xanthine/xanthine oxidase complex formation by 50%.

Biochemical analyses were performed in technical triplicate on a Multiskan FC microplate reader (Thermo Scientific) and analysed with Skanlt software. Beta-esterase was analysed on an LB941 Tristar microplate reader (Berthold Technologies) and analysed with Mikrowin 2000 software.

### 2.8. Proteomic analyses

#### 2.8.1. Midguts dissection

As well as the biochemical analyses, mosquitoes tested for proteomics were not exposed to insecticides.

**Fig 1:**
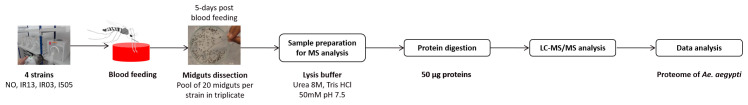
Mains steps of the proteomic experiment.

Proteomic analyses were performed on pools of 20 midguts for the four tested strains (IR03, IR05, IR13 and NO) sampled five days post blood feeding considering a full digestion of the blood. The midguts were dissected on ice, pools of 20 midguts were collected per condition in lysis buffer (Urea 8M, tris HCl 50 mM, pH 7.5) in “Eppendorf™ Protein LoBind Tubes”, and three independent biological replicates were assayed by repeating the experiment three times. The main steps in the analysis of proteins by mass spectrometry are presented in Fig 1.

#### 2.8.2. Sample preparation for MS

Midguts were disrupted by ultrasound (Cup Horn, Sonics & Material) for 20 min with 2 sec pulse on and 2 sec pulse off, at the maximum amplitude and were centrifuged 15 min at 14000g at 4°C. Total protein content in each lysate was analyzed using the Pierce Coomassie Protein assay kit (ThermoFisher™), in 96-well microplates using a microplate reader (MultiSkan Ascent, ThermoLab Systems). Concentration was obtained by comparison with a standard curve of bovine serum albumin using Ascent software (Thermo Labsystems).

#### 2.8.3. Protein digestion

Fifty micrograms of total proteins were reduced in 5 mM TCEP (Sigma − 646547) for 30 min at 26°C and alkylated in 20 mM iodoacetamide (Sigma—I114) for 30 min at 26°C in dark. Proteins were digested with 1 μg rLys-C (Promega—V1671) for 3h30 at 30°C. Samples were then diluted at 1:8 and 0.625 μg of Sequencing Grade Modified Trypsin (Promega—V5111) was added for 8 h at 37°C. The digestion was stop with 0.75% formic acid, and peptides were desalted on reversed phase C18 Sep-Pak Cartridge (Waters—WAT054955). Peptides were eluted twice with elution buffer (50% Acetonitrile (ACN)/ 0.1% Formic acid (FA)). Finally, samples were concentrated using a speed vac (Eppendorf) and then lyophilized. Dried samples were kept at -80°C until mass spectrometry analysis.

#### 2.8.4. LC-MS/MS analysis

LC-MS/MS analysis of digested peptides was performed on an Orbitrap Q Exactive Plus mass spectrometer (Thermo Fisher Scientific, Bremen) coupled to an EASY-nLC 1200 (Thermo Fisher Scientific). A home-made column was used for peptide separation (C_18_ 40 cm capillary column picotip silica emitter tip (75 μm diameter filled with 1.9 μm Reprosil-Pur Basic C_18_-HD resin, (Dr. Maisch GmbH, Ammerbuch-Entringen, Germany)). It was equilibrated and peptide were loaded in solvent A (0.1% FA) at 800 bars. Peptides were separated at 250 nl.min^-1^. Peptides were eluted using a gradient of solvent B (ACN, 0.1% FA) from 3% to 22% in 160 min, 22% to 50% in 70 min, 50% to 90% in 5 min (total length of the chromatographic run was 250 min including high ACN level steps and column regeneration). Mass spectra were acquired in data-dependent acquisition mode with the XCalibur 2.2 software (Thermo Fisher Scientific, Bremen) with automatic switching between MS and MS/MS scans using a top-10 method. MS spectra were acquired at a resolution of 70000 (at *m/z* 400) with a target value of 3 × 10^6^ ions. The scan range was limited from 300 to 1700 *m/z*. Peptide fragmentation was performed using higher-energy collision dissociation (HCD) with the energy set at 27 NCE. Intensity threshold for ions selection was set at 1 × 10^6^ ions with charge exclusion of z = 1 and z > 7. The MS/MS spectra were acquired at a resolution of 17500 (at *m/z* 400). Isolation window was set at 1.6 Th. Dynamic exclusion was employed within 45s.

### 2.9. Data analysis

#### 2.9.1. Insecticide assays

LC_50_ and LC_99_ were calculated by log-probit analysis in R script for bioassay analyses [28]. This script calculates the mortality–dose regression in a generalized linear model, and the quality of the regression is evaluated in a chi-squared test of the observed numbers of dead mosquitoes and the numbers predicted by the regression. The diagnostic concentrations determined from these analyses correspond to twice the LC_99_. LC_50_ and LC_99_ were determined from four replicates for each chemical concentration with NO *Ae*. *aegypti* strain.

#### 2.9.2. Biochemical analyses

Data were tested for normal distribution and homogeneity of variance, and groups were compared by Kruskall-Wallis complemented by a Dunn multiple comparisons test (GraphPad Prism version 7).

#### 2.9.3. Bioinformatics analysis

Acquired MS data were searched using MaxQuant [29, 30] (version 1.5.3.8) (with the Andromeda search engine [31]) against the Uniprot reference proteome of *Ae*. *aegypti* (16650 entries, downloaded the 18^th^ Feb 2017) and a database of chikungunya virus (Frameshift Structural polyprotein, non-structural polyprotein and structural polyprotein, downloaded from Uniprot the 23^rd^ Feb 2018).

The following search parameters were applied: carbamidomethylation of cysteines was set as a fixed modification, oxidation of methionine and protein N-terminal acetylation were set as variable modifications. The mass tolerances in MS and MS/MS were set to 5 ppm and 20 ppm respectively. Maximum peptide charge was set to 7 and 7 amino acids were required as minimum peptide length. A false discovery rate of 1% was set up for both protein and peptide levels.

Quantitative analysis was based on pairwise comparison of LFQ intensities. LFQ were log-transformed (log2). Reverse hits and potential contaminant were removed from the analysis. Proteins with at least 2 peptides (including one unique peptide) were kept for further statistics. LFQ values were normalized by median centering within conditions (normalizeD function of the R package DAPAR [32]). Remaining proteins without any LFQ value in one of both conditions have been considered as proteins quantitatively present in a condition and absent in the other. They have therefore been set aside and considered as differentially abundant proteins. Next, missing values were imputed using the impute.MLE function of the R package imp4p [33]. Statistical testing was conducted using a limma t-test [34] thanks to the R package limma [35]. An adaptive Benjamini-Hochberg procedure was applied on the resulting p-values thanks to the function adjust.p of R package cp4p [36] using the robust method described in [37] to estimate the proportion of true null hypotheses among the set of statistical tests. The proteins associated to an adjusted p-value inferior to a FDR level of 1% have been considered as significantly differentially abundant proteins.

#### 2.9.4. Data availability

The mass spectrometry data have been deposited at the ProteomeXchange Consortium (http://www.proteomexchange.org) [38] via the PRIDE partner repository [39] with the dataset identifier PXD020408.

## 3. Results

### 3.1. Diagnostic concentrations

Diagnostic concentrations may vary between species and no data exist for some insecticides [24]. It was consequently a prerequisite to evaluate the susceptibility of *Ae*. *aegypti* mosquitoes to insecticides. Baselines susceptibility for a selection of nine insecticides from different families were assessed for the susceptible strain (NO). Log-probit analysis allowed us to obtain LD_50_ and LD_99_ values and newly calculated diagnostic concentrations (Table 1). The linear model used fit for all the tested insecticides, with chi-squared values ranging from *p* = 0.803 to *p* = 1, except for permethrin, with *p* = 0.479. Overall, the diagnostic concentrations we obtained for our susceptible reference strain were lower than those obtained for an *Ae*. *aegypti* reference strain in Thailand [40, 41] and closed to those existing in WHO recommendations [24].

**Table 1:** Diagnostic concentrations of the reference strain of *Aedes aegypti* (susceptible strain: New Orleans).

Insecticide	Number of tested mosquitoes	Chi-square (p)	LD50 (%)	95% upper lower limit (%)	LD99 (%)	95% upper lower limit (%)	Diagnostic concentration (%)
**Deltamethrin**	609	0.983	0.002	[0.001–0.002]	0.019	[0.012–0.039]	**0.037**
**α-cypermethrin**	407	0.997	0.003	[0.002–0.004]	0.044	[0.028–0.094]	**0.088**
**Permethrin**	308	0.479	0.066	[0.058–0.074]	0.291	[0.199–0.621]	**0.582**
**Fipronil**	422	0.998	0.051	[0.046–0.055]	0.198	[0.155–0.293]	**0.397**
**Bendiocarb**	408	0.990	0.019	[0.017–0.020]	0.043	[0.037–0.055]	**0.087**
**Propoxur**	405	0.877	0.021	[0.019–0.023]	0.052	[0.042–0.077]	**0.104**
**Malathion**	410	1.000	0.359	[0.350–0.371]	0.478	[0.446–0.536]	**0.957**
**Chlorpyrifos-methyl**	414	0.937	0.063	[0.059–0.068]	0.156	[0.133–0.195]	**0.312**
**Fenitrothion**	613	0.921	0.079	[0.074–0.083]	0.213	[0.184–0.261]	**0.427**

### 3.2. Susceptibility of laboratory strains to several insecticides

#### 3.2.1. Susceptibility to pyrethroids

**Fig 2:**
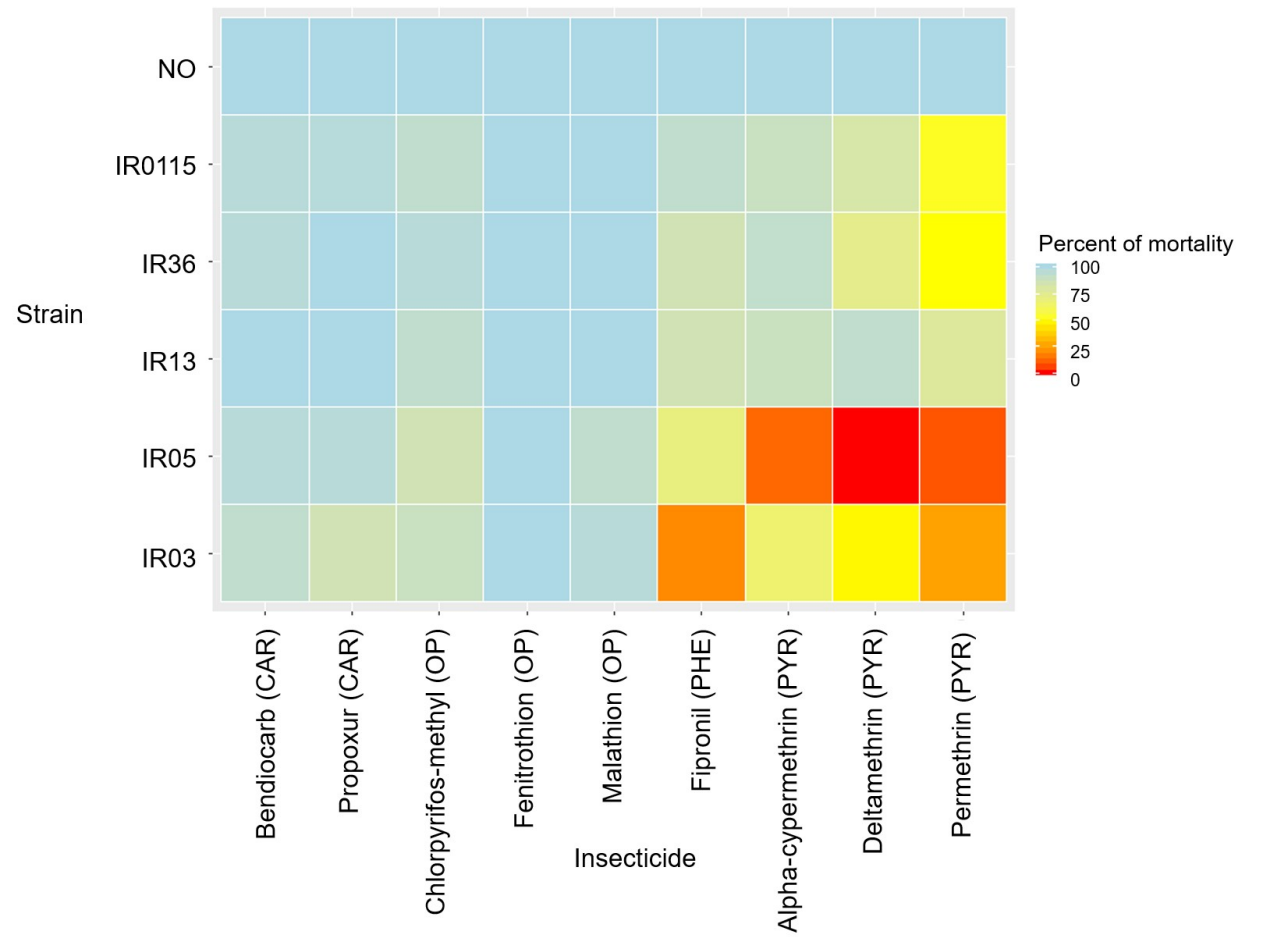
Heat map representing percentage 24-h mortality after exposure to diagnostic dose of several class of insecticides (CAR: Carbamates; OP: Organophosphates; PHE: Phenilpyrazole; PYR: Pyrethroids) for laboratory Aedes aegypti strains: IR13, IR36, IR03, IR05 and IR0115 and New Orleans (NO).

The susceptibility to pyrethroids of adult *Ae*. *aegypti* mosquitoes to the diagnostic concentrations is shown in Fig 2 and S1 Table.

The pattern of 24-h mortality of isofemale strains IR13 and IR36 were similar to those of the original population, IR0115, for all the tested insecticides. All three strains (IR0115, IR13 and IR36) were considered resistant to pyrethroids (represented as mean ± standard deviation), with 87.6 ± 4.5%, 89.6 ± 5.6% and 76.5 ± 2.9% mortality at 24 h for deltamethrin, 90.4 ± 4.9%, 87.3 ± 3.7% and 89.3 ± 8.5% mortality for alpha-cypermethrin and 64.2 ± 13.5%, 81 ± 5.3% and 41.6 ± 7.5% mortality for permethrin, respectively. Strains IR03 and IR05 were more resistant to all the pyrethroids tested than the other strains, with 50.1 ± 9.2% and 2.9 ± 5.8% 24-h mortality with deltamethrin, 60.2 ± 4.2% and 8.7 ± 3.7% mortality with alpha-cypermethrin and 15.5 ± 3.2% and 65 ± 3.2% mortality with permethrin, respectively.

In the meantime, only IR05 exhibited less than 15% 1-h mortality for the three pyrethroids suggesting *kd*-resistance while all the other strains were susceptible with 100% 1-h mortality (S1 Fig).

#### 3.2.2. Susceptibility to organophosphates

The two isofemale strains IR13 and IR36 were susceptible to the three organophosphates tested (malathion, fenitrothion and chlorpyrifos-methyl), with 24-h mortality ≥ 98% (Fig 2).

IR0115 was also susceptible to malathion and fenitrothion (100% mortality) but had possible resistance to chlorpyrifos-methyl, with 94.2 ± 4.9% mortality.

Strains IR03 and IR05 were susceptible to fenitrothion, with 100% mortality, and were resistant to chlorpyrifos-methyl (78.9 ± 6.7% and 78.2 ± 7.1% 24-h mortality, respectively). IR03 exhibited a 24-h mortality rate of 100% with malathion, while IR05 was possible resistant, with a rate of 90.1 ± 2.4%.

#### 3.2.3. Susceptibility to other insecticides

All five tested strains were resistant to the phenylpyrazole fipronil with 24-h mortality rates of 88.5 ± 3.1%, 85.4 ± 4.7%, 80.6 ± 5.3%, 15.5 ± 3.2% and 65 ± 3.2%, respectively.

IR13 and IR36 were susceptible to bendiocarb (100% and 98 ± 2.3% 24-h mortality, respectively) and propoxur (99 ± 1.9% and 100% mortality, respectively). IR0115 appeared to be possibly resistant to bendiocarb, with 97.1 ± 3.7% mortality, but was susceptible to propoxur, with 99.1 ± 1.9% mortality.

The two deltamethrin-selected strains IR03 and IR05 were possibly resistant to bendiocarb (91.2 ± 2.2% and 95.3 ± 3.6% 24-h mortality, respectively) and propoxur (82.8 ± 4.5% and 97.2 ± 3.6% 24-h mortality, respectively).

### 3.3. Genotyping

A total of 294 individuals were genotyped in the allelic discriminating assay for codon 1016 (Table 2). Valine, which is related to deltamethrin susceptibility, was the only amino acid found in the susceptible reference strain NO (N = 48) and in IR13, IR36 and IR03 strains (N = 49, N = 50 and N = 47, respectively). Both valine and isoleucine, which are related to resistance, were found in the original IR0115 population with 90% homozygous wild type (VV) and 10% heterozygous genotype (VI). IR05 exhibited 98% isoleucine (II), which is related to deltamethrin resistance, and 2% of the heterozygous genotype VI (N = 50).

**Table 2:** Coexistence of genotypes of V1016I and F1534C mutations in six strains of Aedes aegypti.

strain	VV/FF^a^	VV/FC^b^	VV/CC^c^	VI/FC^d^	VI/CC^e^	II/CC^f^	Total
**NO**	48	0	0	0	0	0	**48**
**IR13**	23	23	3	0	0	0	**49**
**IR36**	37	13	0	0	0	0	**50**
**IR03**	10	22	15	0	0	0	**47**
**IR05**	0	0	0	0	1	49	**50**
**IR0115**	45	0	0	3	2	0	**50**

^*a*^VV/FF: wild-type VV1016/wild-type FF1534

^*b*^VV/FC: wild-type VV1016/heterozygous FC1534

^*c*^VV/CC: wild-type VV1016/homozygous mutant CC1534

^*d*^VI/FC: heterozygous VI1016/ heterozygous FC1534

^*e*^VI/CC: heterozygous VI1016/ homozygous mutant CC1534

^*f*^II/CC: homozygous mutant II1016/homozygous mutant CC1534

A total of 294 individuals were genotyped in the allelic discriminating assay for codon 1534 (Table 2). Phenylalanine, which is related to deltamethrin susceptibility, was the only amino acid found in the reference strain NO. Both phenylalanine and cysteine were found in IR13 and IR36, in the resistant strain IR03 and in the original population IR0115. Homozygous mutant C1534 were found only in IR13 (6%), IR03 (35%), IR0115 (4%) and IR05 (100%).

### 3.4. Enzymatic activity

**Fig 3:**
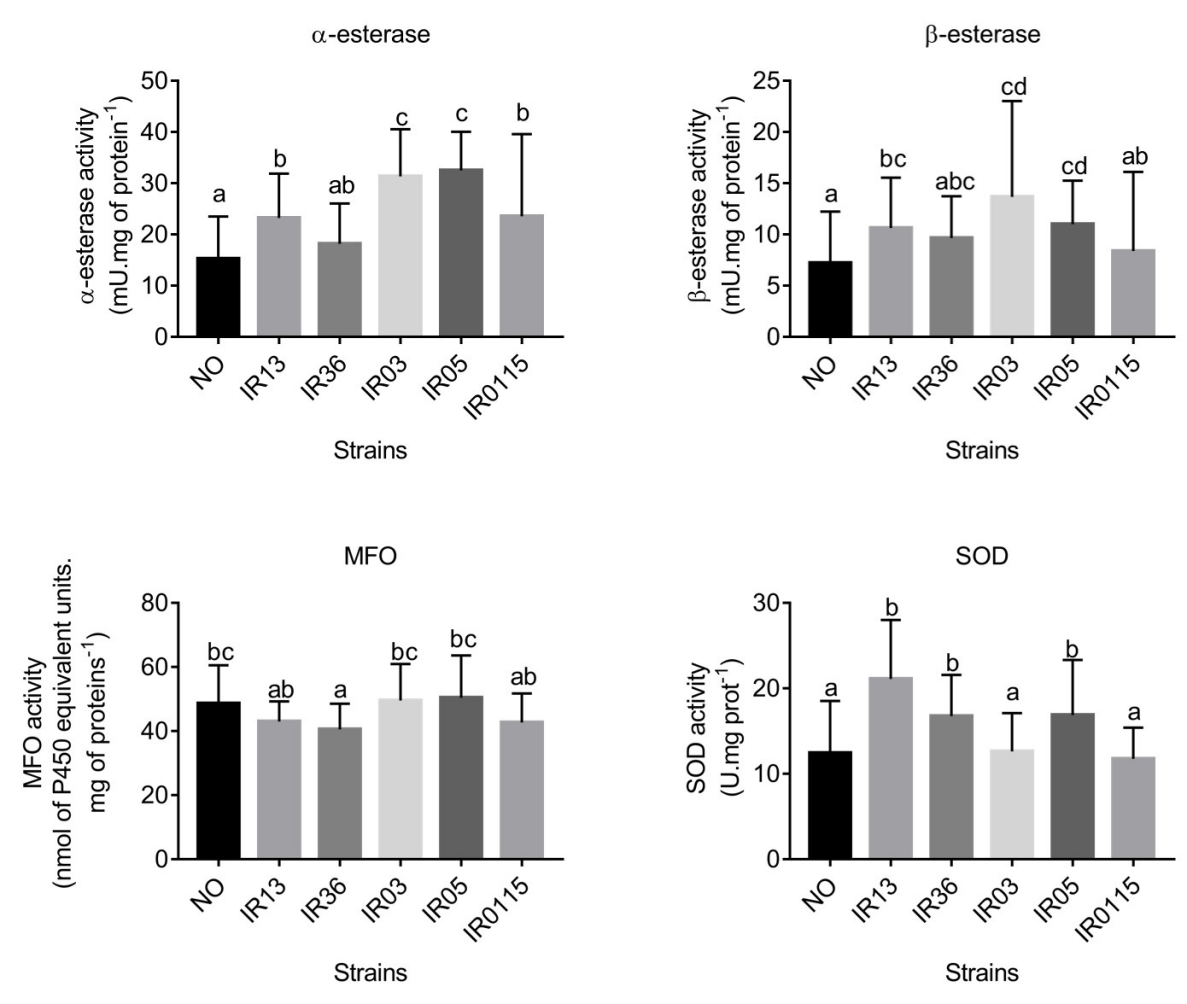
Activities of alpha-esterase, beta-esterase, mixed-function oxidases (MFO) and superoxide dismutase (SOD) in laboratory strains of Aedes aegypti IR13, IR36, IR03, IR05 and IR0115 and in the reference strain New Orleans (NO). Letters indicate significant differences. Values are the means ± SD of 50 individual mosquitoes for each strain.

The activity of alpha-esterase was about 100% greater in IR03 and IR05 than in the reference strain, IR13 and IR36 (Fig 3). The activity of beta-esterase was about 75% greater in IR03 and IR05 than in the reference strain but was not significantly different from that in IR13 and IR36 strains (Fig 3). Mean MFO activity was similar in the reference strain and all the other strains (Fig 3). Mean SOD activity which are enzymes related to resistance was about 75% greater in isofemale strains IR13, IR36 and IR05 than in IR03, IR0115 and the reference strain (Fig 3).

### 3.5. Quantitative proteomics identifies keys protein factors involved in resistance

We chose the most contrasted lineages to run proteomics experiments whose midguts protein content was analyzed by MS. We used this organ because many genes such as CYP450 are expressed in tissues classically involved in xenobiotic metabolism including midgut [42]. Furthermore, it seemed interesting to study the actors involved in resistance to insecticides within this organ, which is also involved during infection by an arbovirus.

A large number of peptides (more than 50723) was detected, which correspond to 4260 identified proteins in total (S2 Table). Venn diagram of identified proteins show a very high reproducibility between samples (S2A Fig). For protein quantification, a high coefficient in Pearson correlation was observed (S2B Fig), demonstrating the good reproducibility between the samples of our proteomic experiment.

**Fig 4:**
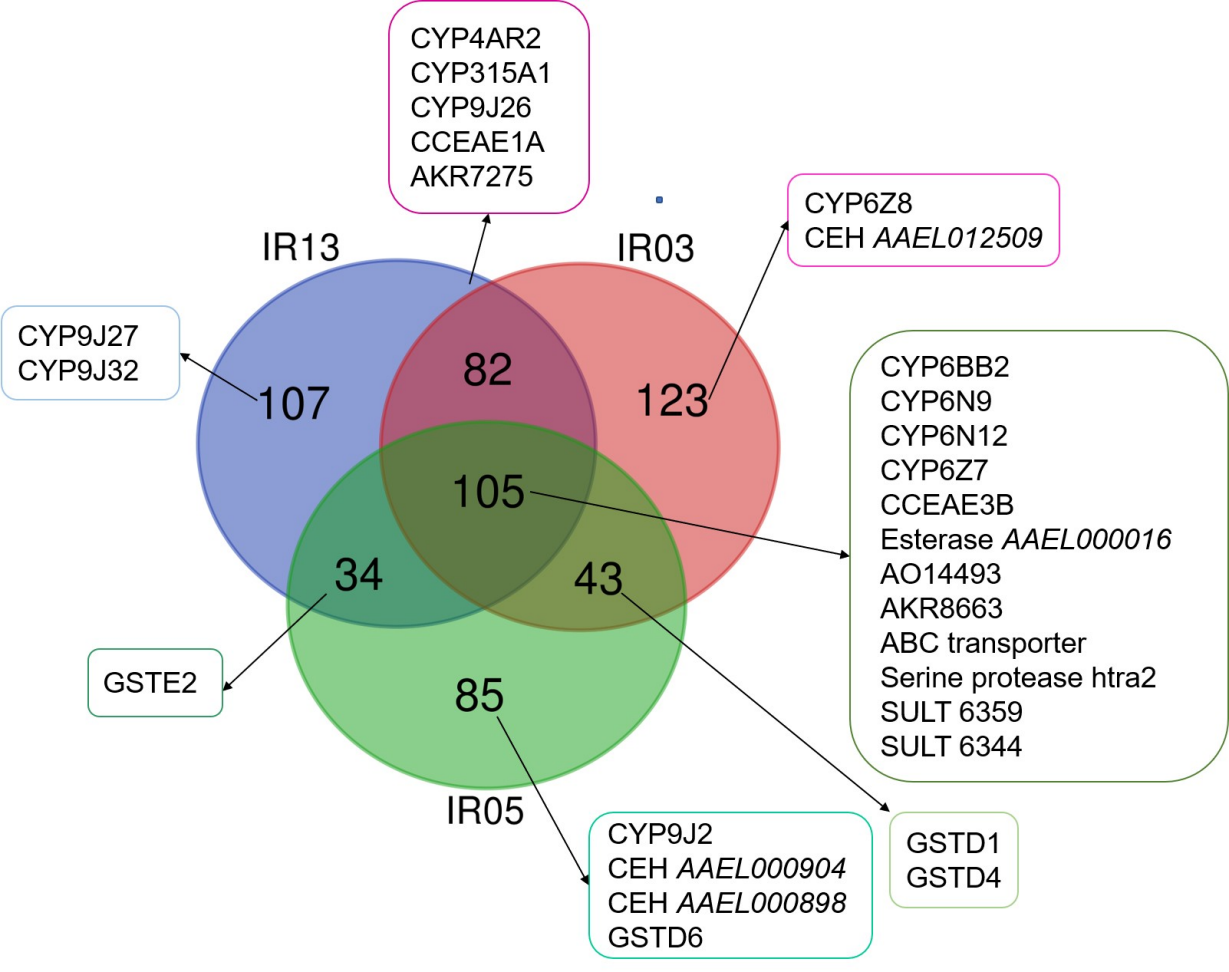
Venn diagram representing the proteins more abundant in either IR13, IR03 or IR05 strains when compared to NO reference strain. The proteins belonging to the cytochrome P450 family (CYP), esterase family (CCE or CEH), GST family, aldo-keto reductase (AKR), serine protease, ABC transporters, aldehyde oxydases (AO) and sulfotransferase (SULT) were highlighted in the different conditions.

We compared the abundance of the proteins in the three strains IR13, IR03 and IR05 to NO reference strain (S3–S5 Tables) We particularly focused on proteins involved in detoxification process or related to insecticide resistance by target specific research on vectorbase (www.vectorbase.org) of cytochrome P450 (CYP), esterases (also named CCE for carboxy/cholinesterase or CEH for carboxyl ester hydrolase), serine protease, ABC transporter, glutathione-S-transferase (GST), aldo-keto reductases (AKR), aldehyde oxydases (AO), sulfotransferase (SULT) and/or enzymes involved in antioxidant response (Fig 4). We observed that several proteins were more abundant or exclusively present in both of the three tested strains compared to NO strain: four proteins from CYP450 family (*CYPBB2*, *CYP6N9*, *CYP6N12*, *CYP6Z7*), two esterases (*CCEAE3B*, *Esterase AAEL000016*), one aldehyde oxydase (*AO14493*), one aldo-keto reductase (*AKR8663*), two sulfotransferases (*SULT6359* and *SULT6344*), one ABC transporter and one serine protease (Fig 4). In addition, there is a combination of proteins likely involved in insecticide resistance in *Ae*. *aegypti* population more abundant in one or more strains compared to the reference strain (Fig 4). *CYP9J27* and *CYP9J32* were more abundant only in IR13. *CYP6Z8* and *CEH AAEL012509* were more abundant only in IR03. *CYP9J2*, two esterases (*CEH AAEL000904* and *CEH AAEL000898*) and *GSTD6* were more abundant in IR05 than in NO. *CYP4AR2*, *CYP315A1*, *CYP9J26*, *CCEAE1A* and one aldo-keto reductase *AKR7275* were more abundant in IR13 and IR03 compared to NO reference strain. *GSTE2* was more abundant only in IR13 and IR05 while *GSTD1* and *GSTD4* were more abundant in IR13 and IR05 compared to NO.

**Fig 5:**
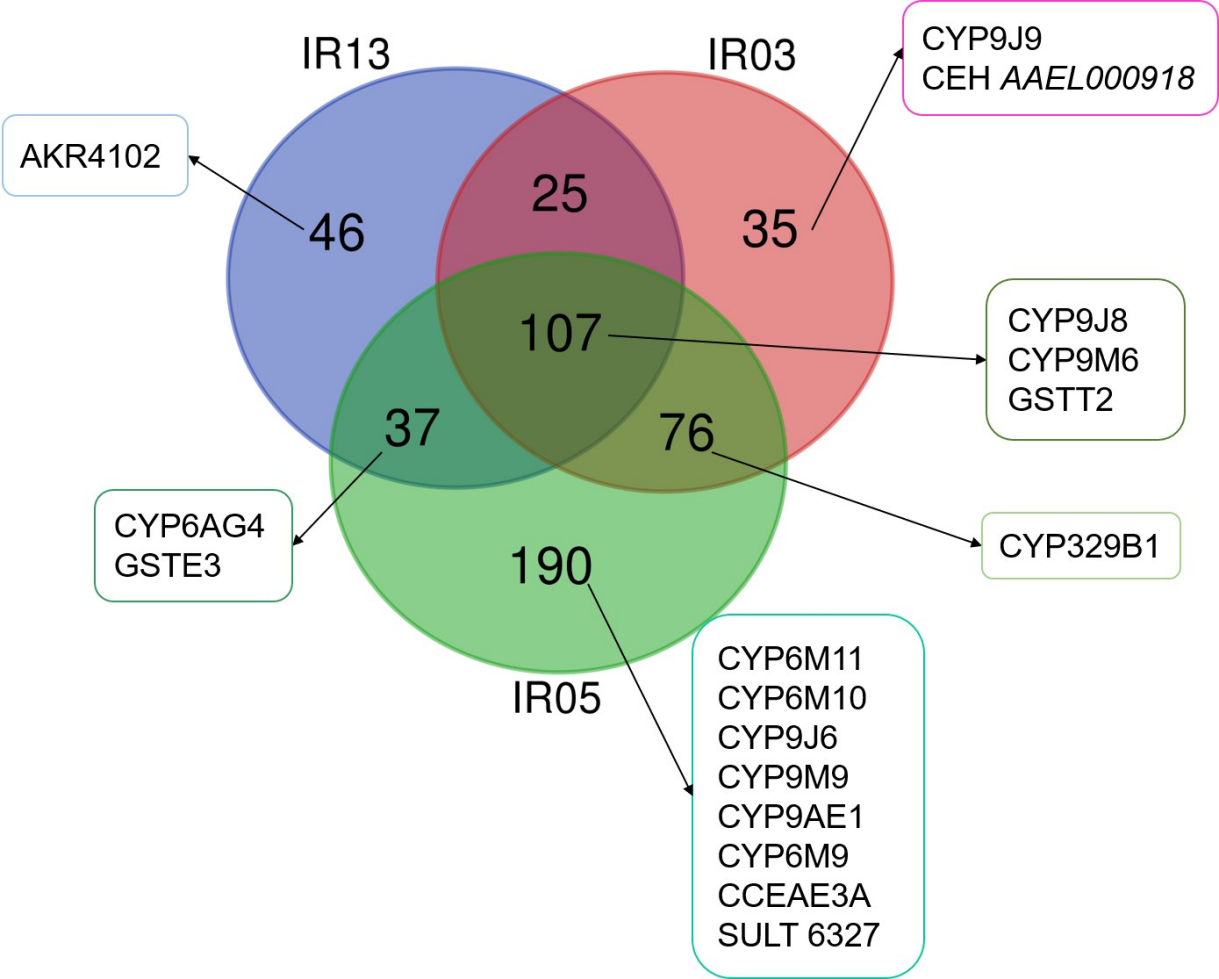
Venn diagram representing the proteins more abundant in NO reference strain when compared to either IR13, IR03 or IR05 strains. The identified proteins belonging to the cytochrome P450 family (CYPs), esterase family (CCE or CEH), GST family, aldo-keto reductase (AKR) and sulfotransferase (SULT) were highlighted in the different conditions.

In addition, some CYP and esterase proteins were also found more abundant in NO than in the other strains (Fig 5) and particularly than in the resistant strain IR05. Indeed, *CYP9J8*, *CYP9M6* and *GSTT2* were more abundant in NO than in the three other strains IR13, IR03 and IR05. The aldo-keto reductase *AKR4102* was more abundant in NO than in IR13. *CYP9J9* and *CEH AAEL000918* were more abundant in NO than IR03 strain. *CYP6M11*, *CYP6M10*, *CYP9J6*, *CYP9M9*, *CYP9AE1*, *CYP6M9*, *CCEAE3A*, *SULT6327* were more abundant in NO than in IRO5 strain. *CYP329B1* was more abundant in NO than in IR03 and IR05 while *CYP6AG4* and *GSTE3* were more abundant in NO than in IR13 and IR05.

## 4. Discussion

### 4.1. Overall phenotype of resistance in isofemale strains

We obtained four isofemale strains from one natural population collected on Ile Royale, an island with low insecticide pressure. Isofemale strains were used to limit interindividual variation and we obtained a panel of phenotypes with different mechanisms involved in insecticide resistance. However, a core of CYPs described to be involved in insecticide resistance were commonly more abundant in three of the strains compared to the reference one suggesting a common metabolic resistance. We hypothesize that during their rearing in the insectary, some strains received deltamethrin treatment that led after several generations to four isofemale strains with different patterns of resistance to deltamethrin and also to other insecticides. Two of the strains, IR13 and IR36, were moderately resistant to deltamethrin, with no *kdr* phenotype and no 1016 mutation, and similar activity of detoxification enzymes to reference strain NO. IR03 and IR05 were highly resistant after deltamethrin pressure. Even though they had the same deltamethrin exposure at larval stages, IR03 and IR05 had different resistance profiles in the genotype of V1016I and F1534C mutations and also different enzyme activities. However, selection pressure could also have played a role on the enzyme panel because of the difference in *kd* mutation at the origin in these two strains. The four isofemale strains were then used as a tool to elucidate the development of multiple resistance and clarify the functions of different mechanisms. All the strains reared in the laboratory were resistant to the three pyrethroids tested (Table 2). The resistance pattern of the two isofemale strains with no selection was overall similar to that of the original strain. Multiple resistance to pyrethroids in all the tested strains is not surprising, as resistance to other insecticides in the same family has been described in mosquitoes [9, 43].

The two pyrethroid-resistant strains (IR03 and IR05) also showed resistance to other classes of insecticides, such as a phenylpyrazoles (fipronil), carbamates (propoxur and bendiocarb) and, to a lesser extent; organophosphates such as malathion and chlorpyrifos-methyl, exhibiting multiple resistance profiles. Our experimental design did not allow us to clearly distinguish whether these strains already had multiple resistance in the field or whether it resulted from deltamethrin selection but we can assess based on other study that deltamethrin selection might have selected more than one mechanism of resistance, suggesting cross-resistance (i.e. resistance to one insecticide conferring resistance to another, even when the insect has not been exposed to both). Cross-resistance between carbamates and pyrethroid has already been described, mainly in the malaria vector *Anopheles gambiae*, through specific P450 detoxification enzymes [44, 45]. Multiple and cross-resistance are common phenomena in *Aedes* mosquitoes submitted to strong insecticide pressure in several regions in the world [4, 10, 46–49]. Resistance to deltamethrin has been described in Brazilian populations of *Ae*. *aegypti* even after pyrethroids had no longer been used for several years, suggesting that some mechanisms of resistance can be maintained even in the absence of pyrethroids [50]. Under laboratory conditions, cross-resistance to pyrethroids was induced after selection with the organophosphate malathion in *Ae*. *aegypti* in Cuba [51], suggesting that resistance to several class of insecticides may involve the same mechanisms of detoxification.

Although the mosquitoes used in this study were collected from an island with low insecticide pressure, one of the hypotheses to explain this cross-resistance to pyrethroids may be found in the past resistance to DDT an organochlorine compound. Moreover, French Guiana has been under insecticide pressure for more than 50 years, which may explain the possible resistant genetic background of *Ae*. *aegypti* populations for chemicals [52].

### 4.2. *Kdr* mutations strongly involved in pyrethroid resistance

Only *Ae*. *aegypti* strain IR05 exhibited the *kdr* phenotype and the resistant 1016 allele, which were totally absent in the other strains. Strong resistance to alpha-cypermethrin was also observed, suggesting the involvement of the V1016I mutation for type II pyrethroids. Strong permethrin resistance was observed in all the strains in our study that exhibited F1534C mutations, suggesting, as already observed in *Ae*. *aegypti* populations in Portugal and Thailand, a greater role of this mutation in resistance to type I pyrethroids [26, 48]. An additive effect to pyrethroid resistance in *Ae*. *aegypti* populations in Asia and South America of a combination of the two *kdr* alleles has already been reported [53–55] and might explain the greater resistance in one of our strains (IR05). It has also been suggested that other point mutations, such as S989P or I1011M/V, may confer high resistance to pyrethroids when present in combination with one or two of the other sodium channel mutations [55–57]. Assessment of these mutations in several populations of *Ae*. *aegypti* [58, 59] did not, however, indicate an association with pyrethroid resistance, particularly deltamethrin. In contrast, F1534C and V1016I have been described as the main mutations responsible for resistance to type I and type II pyrethroids, respectively, in combination or alone [11, 46, 60]. Recently, other mutations in the sodium channel have been described in South American populations of *Ae*. *aegypti*, such as V410L or V1016G [12, 13] and may be tested in our strain in further experiments. Regarding IR05 strain which is *kd*-resistant, our study confirms the strong association between *kdr* and the coexistence of the mutant genotypes I1016I and C1534C which was also observed in *Ae*. *aegypti* from Colombia [61].

### 4.3. Metabolic resistance involves specific CYP450s

We found that the basal activity of non-specific alpha-esterase was greater in the two strains most resistant to deltamethrin than in either the reference or the moderately resistant strains. The basal activity of beta-esterase was higher in the two strains most resistant to deltamethrin than in the reference strain but was not significantly different from the other strains. Although one detoxifying enzyme is not necessarily involved for a particular insecticide, esterases are generally associated with resistance to organophosphates, particularly temephos [18, 46, 62–64] and MFO involved in pyrethroid resistance [14, 16, 17]. Increased alpha-esterase activity could be related to resistance to organophosphates (mainly chlorpyrifos methyl), which we observed only in the two resistant strains IR03 and IR05. Our results are in line with those studies suggesting that alpha-esterase activity is involved in cross-resistance to a variety of insecticides, including pyrethroids, organophosphates and carbamates [8, 16, 65–69].

Several mechanisms, such as increased activity of enzymes belonging to the antioxidant system or to the detoxification process and modification of the respiratory chain and subsequent modification of NADH/NAD+ equilibrium may lead to an increase in reactive oxygen species (ROS) potentially observable by SOD activity. Increased cytochrome P450 activity in insects has been associated with ROS production [70]. In our study, SOD activity was not representative of perturbance of the antioxidant system. Antioxidant mechanisms of defence against insecticides may be more strongly associated with other antioxidant enzymes, namely glutathione *S*-transferase and catalase, as observed in *Anopheles gambiae* resistant to permethrin [71]. Enzyme activity should be assessed not only at baseline but also during exposure of these strains to insecticides to better characterize the mechanisms involved.

The high throughput proteomic analysis performed in this study highlights that the enzyme markers that we observed by biochemistry analyses may not be very specific but may involve particular enzymes such as CYP450s. Even if MFO activity was not significantly different between the tested strains, we observed particular CYPs more or less abundant in the tested strains (IR13, IR03 and IR05) compared to NO. We thus confirmed in our study that the estimation of total enzymatic activity for large enzyme groups such as oxidases is not enough to describe metabolic resistance in so far as specific CYPs may be involved as resistance mechanisms and could not always be detected by biochemical tests. That was suggested in previous molecular studies demonstrating the involvement of CYPs candidate while MFO total activity was not significantly different between susceptible and resistant strains [5, 72, 73]. The specific approach on midguts was chosen for proteomic analyses because detoxification processes are initiated in this organ with high metabolic rates [74]. We observed that only four CYPs (*CYP6N9*, *CYP6N12*, *CYP6Z7*, *CYP6BB2*) were more abundant in the three tested strains (IR05, IR03, IR13) in comparison to NO. These four proteins, members of CYP6 subfamily were already described to be associated with pyrethroid resistance in transcriptomic studies [11, 20]. Our results may confirm the strong role of these proteins in the development of resistance and particularly for *CYP6BB2* and *CYP6Z7* which were described to be directly involved in the metabolization of pyrethroids [75]. Other enzymes such as one ATP-binding cassette transporter (ABC transporter), one aldehyde oxidase, two sulfotransferases, one aldo-keto reductase and one serine protease were more abundant in the resistant strains. ABC transporters belong to a large class of transmembrane proteins, are widely found in insects and play an important role in the transport of xenobiotics and may play a similar role in response to insecticides [76]. Sulfotransferases are known to be involved in phase II detoxification processes and act by adding a sulphate group to various substrates to facilitate their excretion [77]. Sulfotransferases were also described to be associated with temephos resistance in *Ae*. *aegypti* from Martinique Island [68]. Aldehyde oxidases are described to metabolize many xenobiotics in mammals and may also confer a protective role against insecticides [78]. The aldo-keto reductase (AKR) protein superfamily reduce carbonyl substrates and are involved in stress response and detoxification of xenobiotics [79] and were associated in pyrethroid resistance in several populations of *Ae*. *aegypti* [5]. Serine proteases as well as carboxy/cholinesterase *CCEAE3B* were also described to be associated in deltamethrin resistance in transcriptomic studies and may play a role in the degradation of xenobiotics [11]. This panel of proteins more abundant in the strains may be key indicators of resistance to pyrethroids.

However, some CYPs, esterases or GST described to be associated with pyrethroid resistance strains in populations of *Ae*. *aegypti* from Asia or South America [11, 80] were over produced in some of the tested strains compared to the reference such as *CYP9J27*, *CYP9J32*, *GSTE2* for IR13, *CYP6Z8*, *GSTD1*, *GSTD4* for IR03 and *CYP9J2* for IR05. The GSTD1 and GSTD4 are usually associated with temephos resistance and may be relevant for several insecticide resistance [60].

In our study, we also observed that other CYPs and esterases were involved in combination for only two of the resistant strains and may play a role in resistance. This heterogeneity in the abundance of proteins associated with the multiple resistance in the tested strains reflects the difficulty to conclude the role of specific CYPs. However, we observed that each strain had a specific signature concerning CYPs and particularly that NO strain was far different from the isofemale ones (S3 Fig). We have obtained robust candidates insofar as, despite different level of resistance between our strains, some proteins, mostly CYPs have emerged. Moreover, as it was described in several *Ae*. *aegypti* laboratory strains [81], CYPs and *kdr* resistance together confers greater than additive pyrethroid resistance, and CYPs in association with esterase may confer cross resistance to carbamates and organophosphates.

Some CYPs associated with deltamethrin resistance in transcriptomic analyses performed in other studies [11, 20–22] were more abundant in our study in the reference NO strain than in the isofemale resistant strains. This was particularly observed in the most resistant strain IR05 which is *kd-*resistant. While association between *kdr* and high expression of CYPs was described in previous studies to increase resistance [81], we hypothesize that the major role of *kdr* in pyrethroid resistance could have led, during the selection of this strain, to a decrease of the role of some CYPs, resulting in a decrease of their abundance, in comparison with susceptible strains. The proper role of theses CYPs may be elucidated by induction studies in order to better understand which protein is really involved in the response to insecticide exposure and subsequently, to insecticide resistance. Some CYPs may also have been selected during rearing process in our experimentation, but even without exposure to a pesticide, we observed a relative stability of the enzymes involved in resistance in all the strains. To conclude whatever the level of resistance, pressure, or genetics (*kdr* or not) we have a core of proteins involved in resistance that exist.

The complex, multifactorial association between mobilized enzyme families and insecticide resistance depend on the history of insecticide pressure, domestic use of insecticides and the geographical origin and genetic background of the mosquito population. In addition, caution must be exercised regarding gene expression that does not appear to be systematically correlated with enzyme activity. Other detoxification enzymes and other cell components must play a significant role in metabolic resistance. The results we have obtained in our study are in line with those obtained by transcriptomics in other studies, thus reinforcing the idea of molecular markers involved in resistance to insecticides which are essential in the management of resistance. To better understand these markers and their implication, a study of their abundance during the exposure to one or more insecticides is to be undertaken in order to better understand the detoxification cycles that take place within the mosquito.

To conclude, the use midguts in this study could help to better understand the cross mechanisms between insecticide resistance and mosquito infection with arboviruses.

## Supporting information

S1 Fig Representation of the percentage of 1-h mortality after exposure to diagnostic dose of deltamethrin, permethrin and alpha-cypermethrin for laboratory Aedes aegypti strains: IR13, IR36, IR03, IR05 and IR0115 and New Orleans (NO). (TIF)

S2 Fig Reproducibility between Aedes aegypti samples used for mass spectrometry-based (MS) proteomics analysis. (A): This figure shows that the similar numbers of proteins are identified in all samples whatever the condition. A large overlap in the protein composition was observed among samples of a same condition, what shows the good reproducibility of the experiments in term of identification of proteins. (B): This figure displays the pairwise correlation matrix: the Pearson correlation coefficients between each pair of samples was computed using all complete pairs of LFQ intensity values measured in these samples. Because strong correlations are observed (minimum of 0. 924) between all the samples, it shows a strong reproducibility of experiments in term of quantification of proteins. (C): This figure displays a hierarchical clustering of the samples using the Ward method and a Jaccard index based distance after replacing missing values by 1 and observed values by 0. This classification shows that the samples belonging to the same condition are grouped together which means that samples of a same condition have missing values located generally at the same proteins, and that these sets of proteins with missing values are different between conditions.
(TIF)

S3 Fig Representation of the CYPs intensity of Aedes aegypti strains IR13, IR03, IR05 and New Orleans (NO). The heatmap was generated from iBAQ intensity of all CYP protein detected in the proteomic dataset. The HeatMapper tool [82] was used for visualize the heat map and create the dendrogram associated. Complete linkage was choosen for clustering method and measurement was done based on the pearson correlation. Missing value appears in grey while quantitative values are colored according to the scale red-white-green scale as mention in the upper left corner.
(TIF)

S1 Table 24-h mortality after exposure to diagnostic concentrations of nine insecticides for laboratory Aedes aegypti strains: IR13, IR36, IR03, IR05 and IR0115 and New Orleans (NO). (XLSX)

S2 Table Proteomic data concerning all the proteins detected in the midguts of Aedes aegypti mosquitoes. (XLSX)

S3 Table List of proteins that were statistically more or less abundant in IR13 samples compared to NO samples. (XLSX)

S4 Table List of proteins that were statistically more or less abundant in IR03 samples compared to NO samples. (XLSX)

S5 Table List of proteins that were statistically more or less abundant in IR05 samples compared to NO samples. (XLSX)
