# Supplementary figures and images for: CYP450 core involvement in multiple resistance strains of Aedes aegypti from French Guiana highlighted by proteomics, molecular and biochemical studies
Source: PLoS One. 2021 Jan 11;16(1):e0243992. doi: 10.1371/journal.pone.0243992 (PMC7799788; doi:10.1371/journal.pone.0243992)

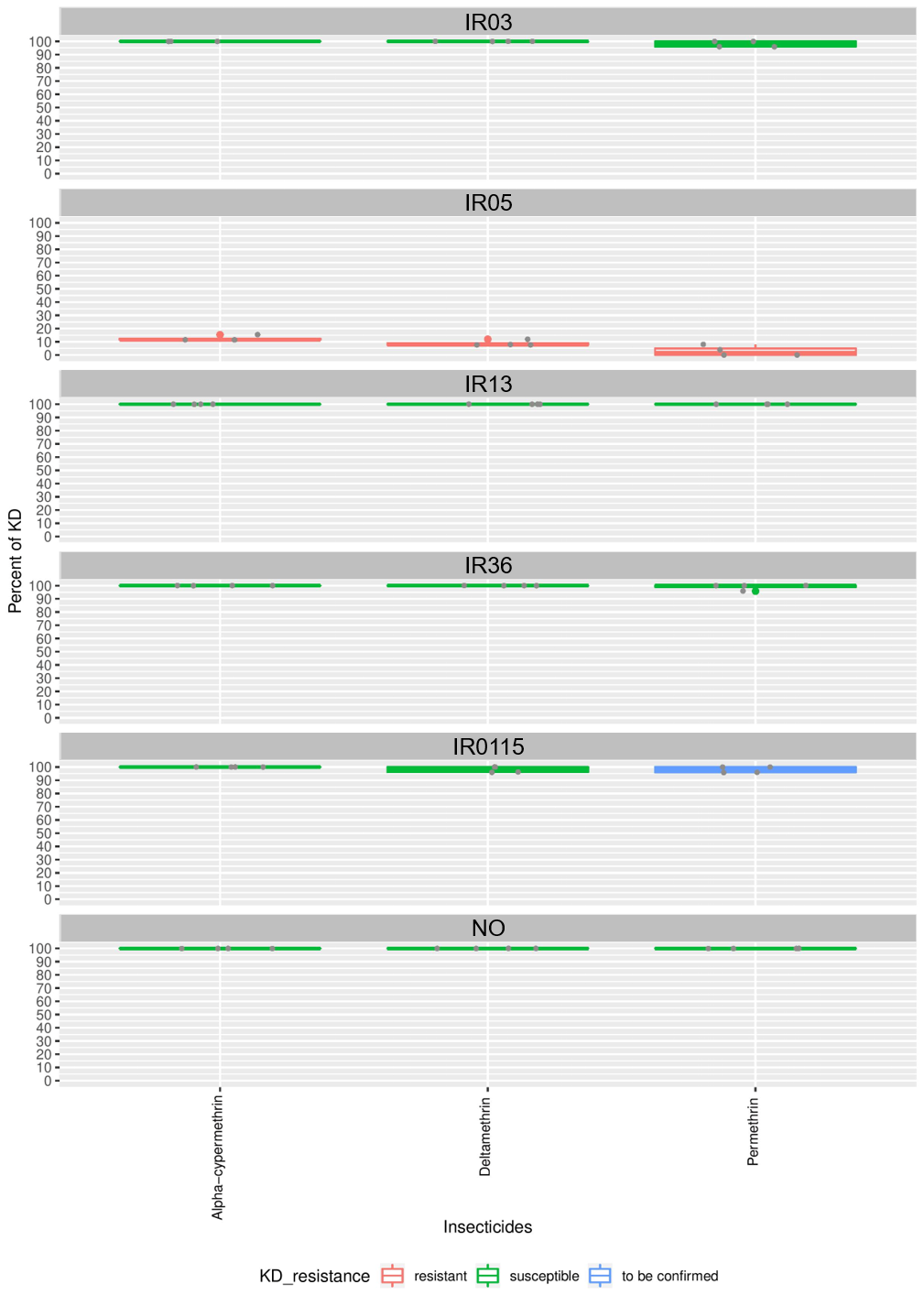

Supplement: S1 Fig — (TIF) [file pone.0243992.s001.tif]

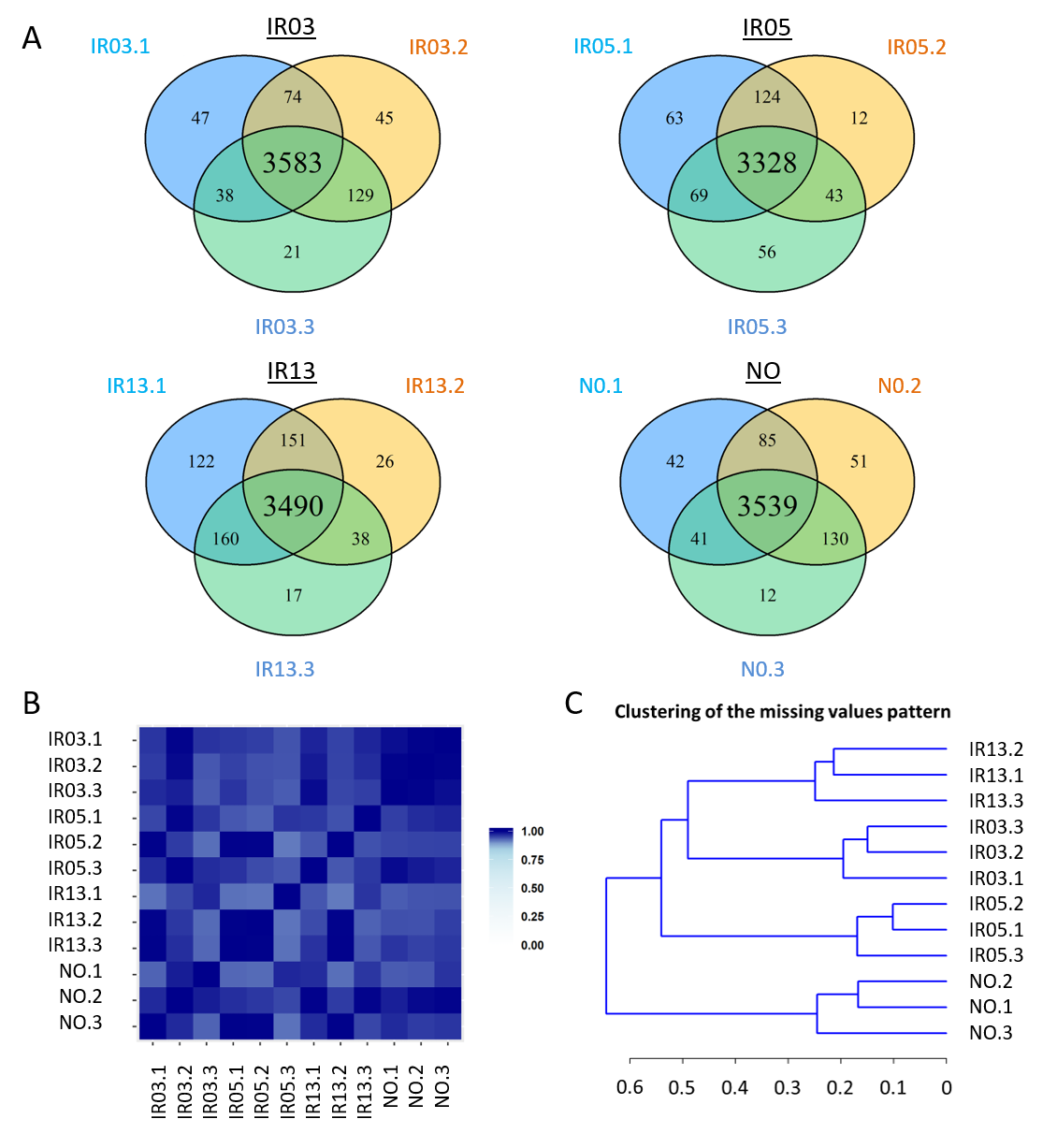

Supplement: S2 Fig — (A): This figure shows that the similar numbers of proteins are identified in all samples whatever the condition. A large overlap in the protein composition was observed among samples of a same condition, what shows the good reproducibility of the experiments in term of identification of proteins. (B): This figure displays the pairwise correlation matrix: the Pearson correlation coefficients between each pair of samples was computed using all complete pairs of LFQ intensity values measured in these samples. Because strong correlations are observed (minimum of 0. 924) between all the samples, it shows a strong reproducibility of experiments in term of quantification of proteins. (C): This figure displays a hierarchical clustering of the samples using the Ward method and a Jaccard index based distance after replacing missing values by 1 and observed values by 0. This classification shows that the samples belonging to the same condition are grouped together which means that samples of a same condition have missing values located generally at the same proteins, and that these sets of proteins with missing values are different between conditions. (TIF) [file pone.0243992.s002.tif]

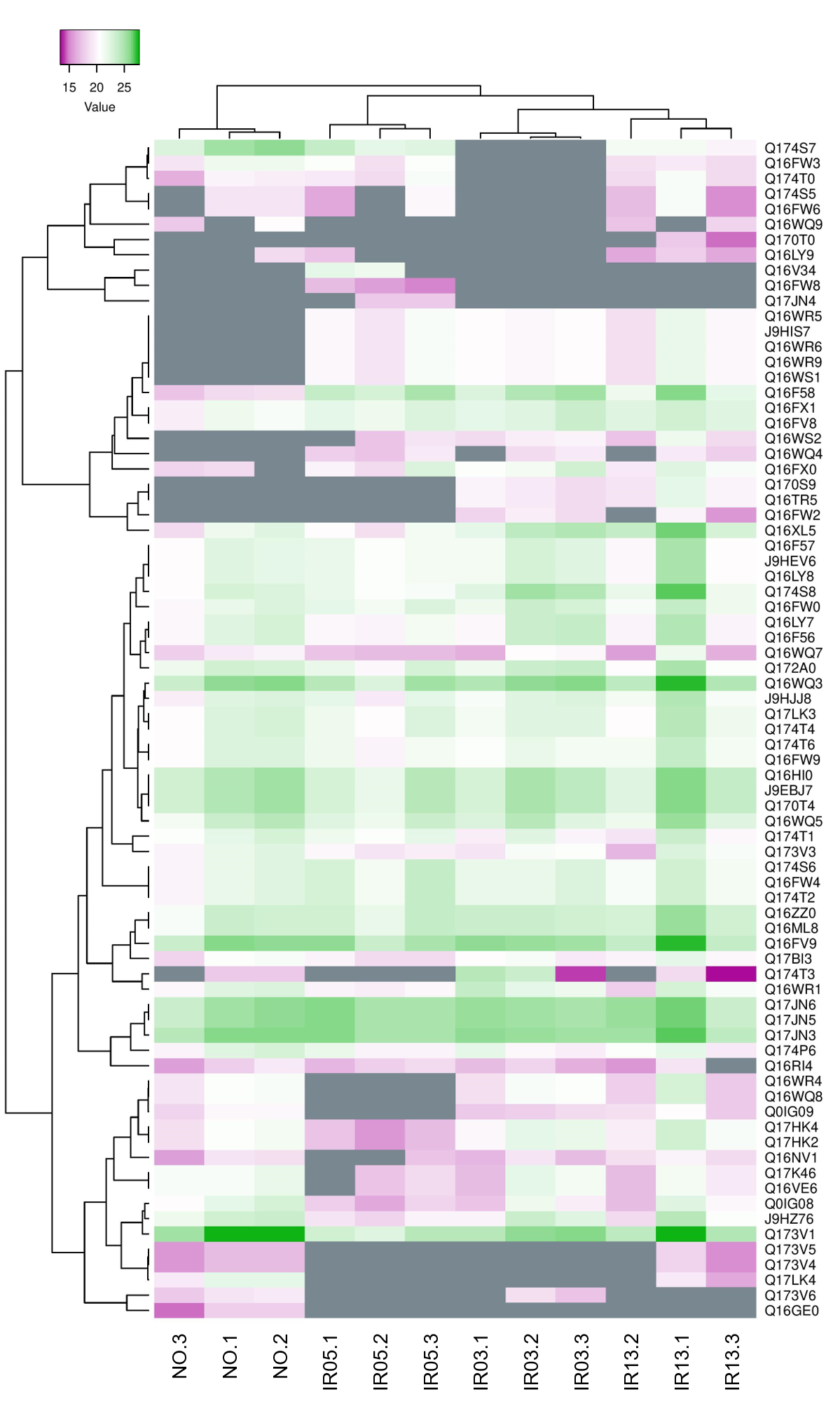

Supplement: S3 Fig — The heatmap was generated from iBAQ intensity of all CYP protein detected in the proteomic dataset. The HeatMapper tool [82] was used for visualize the heat map and create the dendrogram associated. Complete linkage was choosen for clustering method and measurement was done based on the pearson correlation. Missing value appears in grey while quantitative values are colored according to the scale red-white-green scale as mention in the upper left corner. (TIF) [file pone.0243992.s003.tif]
